# *Candida albicans* and *Staphylococcus aureus* Pathogenicity and Polymicrobial Interactions: Lessons beyond Koch’s Postulates

**DOI:** 10.3390/jof5030081

**Published:** 2019-09-04

**Authors:** Olivia A. Todd, Brian M Peters

**Affiliations:** 1 Integrated Program in Biomedical Sciences, College of Graduate Health Sciences, University of Tennessee Health Science Center, Memphis, TN 38163, USA; 2 Department of Clinical Pharmacy and Translational Science, College of Pharmacy, University of Tennessee Health Science Center, Memphis, TN 38163, USA; 3 Department of Microbiology, Immunology, and Biochemistry, College of Medicine, University of Tennessee Health Science Center, Memphis, TN 38163, USA

**Keywords:** *Candida albicans*, *Staphylococcus aureus*, polymicrobial, synergism, co-infection, biofilm, quorum sensing, toxin, fungal-bacterial

## Abstract

While Koch’s Postulates have established rules for microbial pathogenesis that have been extremely beneficial for monomicrobial infections, new studies regarding polymicrobial pathogenesis defy these standards. The explosion of phylogenetic sequence data has revolutionized concepts of microbial interactions on and within the host. However, there remains a paucity of functional follow-up studies to delineate mechanisms driven by such interactions and how they shape health or disease. That said, one particular microbial pairing, the fungal opportunist *Candida albicans* and the bacterial pathogen *Staphylococcus aureus*, has received much attention over the last decade. Therefore, the objective of this review is to discuss the multi-faceted mechanisms employed by these two ubiquitous human pathogens during polymicrobial growth, including how they: establish and persist in inter-Kingdom biofilms, tolerate antimicrobial therapy, co-invade host tissue, exacerbate quorum sensing and staphylococcal toxin production, and elicit infectious synergism. Commentary regarding new challenges and remaining questions related to future discovery of this fascinating fungal–bacterial interaction is also provided.

## 1. Challenging Koch’s Postulates: Polymicrobial Infections

In 1890, Robert Koch published one of the seminal ideologies of modern microbial pathogenesis, now commonly referred to as “Koch’s Postulates”. In order to demonstrate causation between microbe and disease, four straightforward principles were proposed: 1) the microbe must be found in all cases of the disease, but not in healthy organisms, 2) the microbe must be isolated from the diseased individual and grown in pure culture, 3) the isolated organism must cause disease in a healthy individual, and 4) the microbe must be reisolated and reidentified as the original causative agent [1]. Despite the frequent violation of postulate 1, where healthy individuals often asymptomatically carry opportunistic microbes, Koch’s postulates have largely led to a global understanding of disease pathogenesis through the prism of a monomicrobial infection model. However, we now know that microbes rarely exist as single species but are often part of complex polymicrobial communities consisting of bacteria, fungi, viruses, and protozoans. Therefore, it is logical to hypothesize that, at times, interactions between these various microbes and the resulting modulation of gene and protein expression profiles drive disease onset and outcome. Thus, it is difficult to satisfy postulates 2 and 3, given that the existence of multiple microbes may be required to elicit similar infectious outcome. Moreover, the complex interplay between host immune status and genetic composition may confer susceptibility to one individual but not recapitulation of the disease in a second (violation of postulates 3 and 4). While Koch’s Postulates have served the field of microbiology very well in its early stages, their utility in helping to understand the pathogenesis of polymicrobial infections is limited. Herein, we describe various interactions employed by polymicrobial communities and focus on the fungal pathogen *Candida albicans* and the bacterium *Staphylococcus aureus* as prototypical organisms in understanding mechanisms driving exacerbated outcomes during co-infection.

## 2. Interkingdom Interactions and Polymicrobial Biofilms

Bacteria and fungi often occupy the same ecological and biological niches, existing and interacting in various ways to persist, propagate, and prosper. While microbes certainly exist as free-living forms, a majority of microbial life exists as sessile communities coined “biofilms”. Biofilms are three-dimensional, structurally complex communities of one or more microbe, encased in an extracellular matrix consisting largely of polysaccharides [2]. These structures can be formed on both biotic or abiotic surfaces and promote microbial consortia development. Biofilms allow for enhanced adhesion to body surfaces, protection from environmental stressors and the host immune system, and often result in increased tolerance to antimicrobial agents. Due to these properties, biofilms are important sources of infection, and the capacity to form a biofilm is considered an important virulence determinant.

With the advent of high-throughput genome sequencing, our knowledge regarding the distinctive biodiversity existing at specific body sites has expanded exponentially. For example, the oral cavity harbors over 700 unique species, the skin is home to approximately 1000 species, and the gut maintains an incredible biomass composed of greater than 1000 species [3,4]. Given the constrained biological space these organisms share, physical, metabolic, secreted, and environmental mechanisms are bound to shape their interaction. There are five general types of interactions observed within ecological relationships: competition, predation, commensalism, parasitism, and mutualism [5]. These categories can be further simplified by delineating whether the interaction is symbiotic or antagonistic.

Symbiotic relationships can be mutually beneficial for both microbes, favor one microbe, or simply result in a neutral relationship. As mentioned above, the oral cavity is a dynamic environment that plays host to numerous biofilm communities that can be formed on both the mucosa and the tooth surfaces. While a number of different bacterial species can be found in the mouth, *Candida* species are the dominant fungus of the oral mycobiome [6]. *Candida* sp. interacts with *Porphyromonas gingivalis*, a Gram-negative anaerobic bacterium that is commonly associated with the progression of periodontal disease [7]. Bartnicka et al. described the role of *C. albicans* biofilms in establishing an anoxic environment in which *P. gingivalis* can robustly proliferate under such anaerobic conditions, presumably driving gingival inflammation [8]. Similarly, *C. albicans* also demonstrates a mutualistic relationship with *Streptococcus mutans*—a common causative agent of dental caries. *S. mutans* readily metabolizes dietary sucrose into glucose, which is further rapidly metabolized by *C. albicans*, generating a persistent acidogenic-aciduric microenvironment that promotes synergistic tooth demineralization and caries formation [9,10,11,12].

In a competitive relationship, organisms compete for nutrients and resources within a shared environment. An example of this kind of antagonistic relationship is seen between *Pseudomonas aeruginosa*, a Gram-negative bacterium, and *Rhizopus microsporus*, an environmental fungus responsible for a majority of mucormycosis cases. Kousser et al. showed that these two microbes compete for iron within wounds; specifically, *P. aeruginosa* secretes siderophores to take up iron, resulting in inhibition of *R. microsporus* growth and germination [13]. Although mediated through a different mechanism, *P. aeruginosa* also displays antagonism against *C. albicans* by binding to the fungus and secreting toxic phenazine compounds and homoserine lactones that are capable of killing the fungus and repressing filamentation, respectively [14,15]. Multi-faceted relationships (such as those described above) likely frequently occur but on a much grander scale, given the complexity of consortia on environmental and host surfaces. These interactions undoubtedly shape microbial physiology with significant consequences for both the microbe and host.

## 3. Getting the Lines Crossed: Quorum Sensing and Intermicrobial Communication

Interkingdom cross-talk is another component of the interactions between communities of bacteria and fungi, which is mediated through recognition of signals as part of quorum sensing (QS). QS is a density-dependent communicative signaling system that regulates coordinated gene expression within a population [16]. The discovery of QS, first in bacteria and later in lower eukaryotes, such as fungi, reformed the idea that microbes act independently, even when in a community, and are actually quite social [16]. QS is evolutionarily useful for microbes by sensing the changing environment and adapting for the good of the community, including aiding in the acquisition and the sharing of nutrients, tolerance to stress and antimicrobials, adaptation to ecological niches, as well as enhancement of virulence in response to other microbial or host organisms. In bacteria, the QS system relies on an accumulated signal molecule that is produced and recognized by cells, and it is only at certain concentrations of this signal that gene expression is modulated. Many of the signaling molecules are unique to specific species and sometimes even to certain clades within species. For example, there exist four divergent *agr* (accessory gene regulator) quorum sensing systems in *Staphylococcus aureus* that produce different signaling molecules (AIP-I, II, III, and IV). These signals, although structurally very similar, exhibit cross-inhibition, or interference, to the other *agr* types. This *agr* interference leads to inhibition of transcription of the *agr* locus of another type, perhaps providing a competitive advantage for the dominant strain [17]. Conversely, there is some evidence of degenerate signals and promiscuous receptors, allowing for interspecies cross-talk [18]. One study looked at the selectivity of QS receptors across a number of bacterial species and discovered a range of recognition of receptors to non-native quorum signals. Quantitative scoring of interactions took into account the sensitivity of the receptor to the signal as well as the degree of activation upon recognition. The RhlR QS receptor in *P. aeruginosa* is extremely selective for its own quorum signal and showed the highest score in these experiments. On the other side of the spectrum, the Btar2 receptor of *Burkholderia thailandensis* responded strongly to several different QS signal molecules, including those from *P. aeruginosa* and *Vibrio fischeri*. Based on these observations, the Btar2 receptor was defined as promiscuous [18]. In addition to bacteria, fungi also undergo QS [19]. A large body of research exists focusing on the *C. albicans* QS molecule farnesol, a byproduct of sterol synthesis that represses filamentation without disrupting growth rate [20,21]. Interestingly, farnesol also impacts susceptibility to antimicrobials or cell signaling against several bacterial species, including *S. aureus* and *P. aeruginosa* [22,23,24,25]. At high concentrations, farnesol prevents *S. aureus* biofilm formation partially by damaging the bacterial membrane, and it dose-dependently inhibits production of the carotenoid pigment staphyloxanthin, conferring paradoxical resistance to oxidative stressors by increasing biosynthesis of anti-oxidant enzymes [26,27]. While quorum sensing systems exist in other fungal species, the specific molecules have not been identified but appear to be linked to control of dimorphism [28].

**Figure 1:**
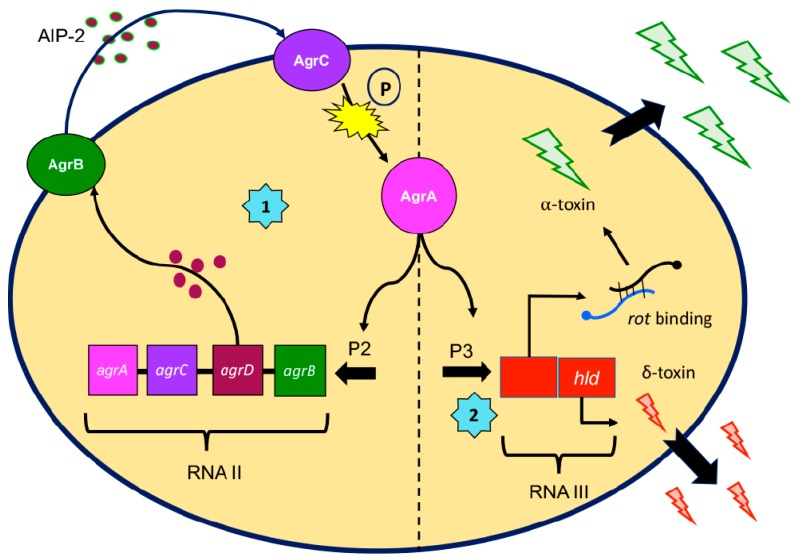
Schematic of the *agr* quorum sensing system in *Staphylococcus aureus*. AgrA is the response regulator in a two-component system that acts as a transcription factor, modulating toxin production. **(1)** AgrA activates transcription from the P2 promoter, driving expression of RNAII, an operon consisting of four *agr* genes. AgrB is a membrane-bound permease that processes AgrD, a pre-signal peptide, and releases it as AIP-2 (auto-inducing peptide 2). AIP-2 is sensed by AgrC, a membrane-bound histidine kinase that is part of the two-component signaling system. AgrC phosphorylates AgrA, activating it, leading to a positive feedback loop. **(2)** Activated AgrA also drives transcription from the P3 promoter, driving expression of RNA III, the effector of the QS system. RNA III directly encodes for delta toxin (*hld*) and also binds to repressor of toxin (*rot*) transcript, allowing for toxin production by inhibiting *rot* translation. RNAIII also binds to a number of adhesin-related genes to similarly block their translation.

The most well-described and studied bacterial QS system in *S. aureus* is the accessory gene regulatory (*agr*) system [16,29,30,31] (see Figure 1 for schematic). It consists of two divergent promoters, P2 and P3, that drive expression of two separate transcripts, RNAII and RNAIII, respectively. RNAII encodes for four genes, *agrA*, *agrB*, *agrC*, and *agrD*. AgrA and AgrC make up a two-component system where AgrA is the response regulator that, when phosphorylated by the membrane-bound histidine kinase AgrC, activates the P2 promoter. AgrD is the pre-signal peptide that is modified and secreted through the membrane-bound AgrB as the mature autoinducing peptide 2 (AIP-2). AIP-2 is then recognized by AgrC, completing the signaling circuit. Alternatively, AgrA can also activate the P3 promoter, driving expression of RNAIII—the effector of the quorum sensing system. RNAIII directly encodes for δ-toxin at its 5’ end. The 3’ end contains a consensus sequence that is complementary to the 5’ end of a number of staphylococcal adhesin genes and, when in complex with such transcripts, prevents their translation by masking the Shine-Dalgarno ribosomal binding site. Via this same mechanism, RNAIII also inhibits the translation of the repressor of toxin (*rot*) transcriptional regulator, allowing for increased production of toxins. Thus, QS signals (such as those produced by *agr* system) are imperative in facilitating information exchange between microbial self and non-self.

## 4. *Candida albicans* and *Staphylococcus aureus*: Co-Conspirators

The remainder of this review focuses on *C. albicans*–*S. aureus* interactions within the context of polymicrobial infections and with a specific emphasis on the mechanisms that contribute to enhanced pathogenicity of such co-infections. *C. albicans* is a polymorphic fungus that exists as an opportunistic pathogen, colonizing the gut and the mucosa of humans, and is one of the most prevalent human fungal pathogens [32]. The capacity to transition between yeast and invasive hyphal morphologies is considered to be its key virulence attribute. The shift from commensal to pathogen is usually due to changes in the immune status of the host. *C. albicans* can cause superficial infections (e.g., candidiasis) of mucosal sites such as the mouth and the vagina [33]. Oral and vaginal candidiasis are the result of an overgrowth of the yeast and are associated with robust inflammation of the mucosal surface [33,34]. A more serious infection is candidemia, a systemic infection where *Candida* invades local tissue, accesses the vasculature, and disseminates throughout the body via the bloodstream.

*S. aureus* is a Gram-positive coccus bacterium that can cause a variety of different diseases ranging from skin and soft-tissue infections to severe bacteremia and sepsis [35]. The pathogenicity of *S. aureus* is primarily mediated through toxin production, which is intimately linked to its quorum sensing system, described in detail above. *S. aureus* produces a number of toxins: the membrane-damaging and cytolytic toxins ⍺- and δ-toxin, Panton–Valentine leukocidin (PVL), and phenol-soluble modulins (PSMs) and the superantigen toxic shock syndrome toxin (TSST), among numerous others [36]. Aside from damage-inducing toxin production, *S. aureus* can also perturb hemostasis through manipulation of the clotting cascade, resist high levels of antimicrobials due to robust biofilm formation, and avoid clearance by immune cells through capsular polysaccharide production [37,38,39]. The multi-faceted and redundant nature of *S. aureus* virulence makes it one of the most formidable human pathogens.

While *C. albicans* and *S. aureus* cause significant morbidity and mortality independently, these microbes are also commonly found together at various body sites and are implicated in a variety of diseases, including cystic fibrosis, ventilator-associated pneumonia, urinary tract infections, superinfection of burn wounds, denture stomatitis, and keratitis [40,41,42,43]. Within the past decade, several in vitro and in vivo animal models have revealed interesting clues as to how these pathogens may cooperate within the host to exacerbate pathogenicity and disease.

### 4.1. Fungal-Bacterial Biofilms and Altered Drug Tolerance

**Figure 2:**
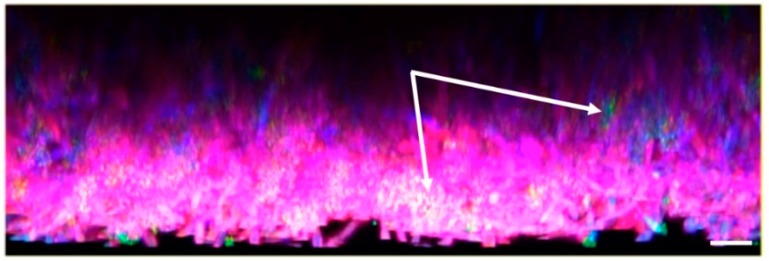
In vitro polymicrobial biofilm formation by *C. albicans* and *S. aureus*. The images demonstrates *S. aureus* (green/merged, white arrows) attached throughout the biofilm and along the hyphal filaments of *C. albicans* (blue). The extracellular matrix (red) largely encases a majority of the staphylococci. **Methods:** A polymicrobial biofilm was formed in vitro in RPMI-1640 medium with *C. albicans* (strain SC5314) and *S. aureus* (strain M2) using 1 × 10^6^ CFU of each microbe to inoculate a Permanox chamber slide for 24 h at 37 °C. Biofilms were washed with sterile saline to remove non-adherent cells, fixed in 4% formalin, and stained with a cocktail containing calcofluor white (50 µg/mL), Concanavalin A-Texas Red (50 µg/mL), and Syto9 (1.67 µM). Images were captured using 405 nm, 488 nm, and 565 nm lasers and DAPI, FITC, and Texas Red filter sets with a Zeis 510 confocal scanning laser microscope. Corresponding Z-stacks were constructed using packaged Zeis software depicting a side view. Scale bar represents 20 µm.

Polymicrobial infections with *C. albicans* and *S. aureus* are common, due in part to shared niches within the body, including co-isolation from skin, axillae, vagina, pharynx, nasal passages, and oral mucosa [44]. *C. albicans* and *S. aureus* both have the ability to form biofilms and thus are commonly found growing in polymicrobial biofilms on indwelling medical devices, such as catheters (Figure 2). These biofilms are difficult to treat with antimicrobials, as the complex structure of the biofilm protects the organisms by impeding drug permeability and immune cell access. Unfortunately, treatment often involves replacing the catheter, which can be life-threatening in patients with limited options for catheter reinsertion.

**Figure 3:**
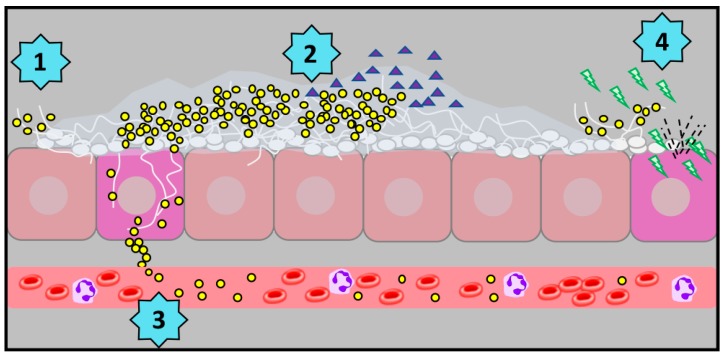
Schematic of *C. albicans*–*S. aureus* interactions. **(1)** *S. aureus* preferentially attaches to the hyphal filaments of *C. albicans* via binding of the candidal adhesin Als3p. **(2)** Encasement of *S. aureus* in fungal biofilm matrix components (including β-1,3-glucan) impairs penetration of antibiotics (purple triangle) by sequestration of drug. The *C. albicans* QS molecule farnesol also upregulates drug efflux pumps in *S. aureus* to enhance tolerance to antibacterials. **(3)** *S. aureus* is able to gains access into subepithelial spaces by “hitchhiking” onto the invasive *C. albicans* hyphae. *S. aureus* may also then disseminate to distant sites (including the kidneys) following co-invasion via the bloodstream. **(4)** *C. albicans* enhances the staphylococcal *agr* QS system, ultimately leading to elevated levels of ⍺-toxin (green bolts), cell damage, and synergistic lethality during polymicrobial IAI.

Among the earliest work to investigate the development of polymicrobial biofilms by these species revealed that large staphylococcal aggregates formed around hyphal filaments of *C. albicans*, and that *S. aureus* preferentially favored binding to these hyphal filaments as compared to round yeast cells [45,46] (Figure 3). Work by Harriott and Noverr demonstrated that *S. aureus* displayed tremendous tolerance to vancomycin [over 1000-fold higher than the planktonic minimal inhibitory concentration (MIC)] during biofilm growth with *C. albicans*. Follow-up studies revealed that viable *C. albicans* was required for this phenotype, and that *S. aureus* became coated with dense extracellular material during polymicrobial growth [45]. Coating of *S. aureus* with isolated fungal matrix polysaccharides (as evidenced by increased concanavalin A staining) revealed that elevated antimicrobial tolerance was driven by encasement of bacteria by the dense fungal extracellular meshwork. Additional studies revealed that *C. albicans* mutants (e.g., *efg1*Δ/Δ/*cph1*Δ/Δ) unable to adhere to the substratrum due to hyphal growth defects were unable to augment vancomycin tolerance in *S. aureus* [47]. Global or specific genetic deletion of adhesins did not seemingly impact vancomycin tolerance profiles during co-culture. Newer work by Kong et al. using both genetic and enzymatic approaches to modulate matrix components coupled with fluorescence microscopy-based drug diffusion assays identified the fungal polysaccharide β-1,3-glucan as the key moiety impeding vancomycin penetration of the biofilm structure [48]. This phenomenon of enhanced staphylococcal drug tolerance during polymicrobial biofilm formation is not vancomycin-specific, as reduced susceptibility to doxycycline, nafcillin, and oxacillin has also been observed [48]. Moreover, the fungal quorum sensing molecule farnesol referred to above was shown to activate staphylococcal drug efflux pumps, enhancing recalcitrance to several antibacterial drugs [48]. Thus, it is clear that co-culture of these organisms can drastically alter phenotypic outcome with respect to drug tolerance and biofilm architecture (Figure 3).

### 4.2. Enhanced Pathogenicity in the Oral Cavity and at a Distance

A major virulence factor of *C. albicans* is the ability to switch from yeast to hyphae. The yeast form is suited for dissemination and initial seeding during infections, while the hyphal form is crucial for tissue penetration and immune evasion and is associated with upregulation of other virulence factors. One of these virulence factors is the production of surface adhesins. Peters et al. demonstrated that *S. aureus* binds to *C. albicans* hyphae through interaction with the candidal adhesin Als3p [49]. Confocal fluorescence microscopy of *C. albicans*–*S. aureus* biofilms qualitatively showed decreased association of *S. aureus* to *C. albicans* hyphae lacking Als3p as compared to wild-type. The strength of this interaction was quantified by measuring the adhesion forces between *C. albicans* and *S. aureus* by atomic force microscopy, confirming weaker binding when Als3p was genetically deleted [49]. The importance of Als3p binding by *S. aureus* during infection was demonstrated in an oral model of polymicrobial infection [50]. When the oral cavity was infected with *C. albicans* and *S. aureus*, immunocompromised mice developed systemic infections with high microbial burdens in the kidneys and elevated mortality. Mono-infected mice (*C. albicans* or *S. aureus* alone) as well as mice infected with *S. aureus* and *C. albicans als3*Δ/Δ did not develop systemic infection [50]. Similar results were found when *S. aureus* was co-inoculated with the hypha-defective *efg1*Δ/Δ/*cph1*Δ/Δ mutant of *C. albicans* [51]. Thus, both hyphae and Als3p are required for *S. aureus* to disseminate from the oral cavity. The staphylococcal receptors required for robust binding to Als3p are likely multifactorial, although *S. aureus* mutants defective for fibronectin binding protein B (Δ*fnb*), staphylococcal surface protein F (Δ*sasF*), and autolysin (Δ*atl*) demonstrated reduced capacity to bind hyphae in vitro [50]. Cumulatively, these results led to development of a hypothetical model in which *S. aureus* could “hitchhike” onto the invasive filaments of *C. albicans*, gain access to submucosal tissue, and disseminate to distant sites (Figure 3). Fluorescence in-situ hybridization images of tongue tissue from co-infected mice supported this hypothesis, as hyphae embedded deep into the epithelium were found surrounded by attached staphylococci [50].

*C. albicans* and *S. aureus* are both implicated in denture stomatitis, where the oral mucosa is inflamed and lesions are formed [36]. A study by Baena-Monroy et al. examined saliva and culture swabs of denture surfaces from over 100 subjects fitted with dentures [52]. Using culture-based techniques, they found that *C. albicans* and *S. aureus* could be recovered from the oral mucosa and the denture surfaces of both denture stomatitis patients and healthy controls. However, increased levels of *C. albicans* was recovered from the denture surface, while *S. aureus* was found predominantly in the oral mucosa of denture stomatitis cases. These results suggest that *C. albicans* may facilitate colonization of *S. aureus* during denture stomatitis and enable staphylococcal superinfection via a mechanism that could be explained by the aforementioned co-invasion hypothesis.

### 4.3. Polymicrobial Intra-Abdominal Infection and Lethal Synergism

Intra-abdominal infections (IAI) are a collection of a spectrum of diseases characterized by microbial infection within the abdominal cavity and resulting inflammation of the peritoneum. The majority of these infections are caused by a breach of the gastrointestinal tract epithelium, facilitating the invasion of microbes [53]. IAI are the second most common cause of sepsis in intensive care unit (ICU) patients and typically have a high mortality rate [54]. Polymicrobial IAI are correlated with a more severe disease state and higher rate of mortality, specifically when a fungal pathogen is involved, with mortality reaching 80% [55]. This is in contrast to IAI caused by bacteria only, which display associated mortality rates of up to 30% [56]. *C. albicans* and *S. aureus* are among the top most commonly isolated organisms during IAI [55]. Dissemination of microbes from the peritoneal cavity leads to systemic infection and can progress to sepsis. Sepsis is caused by the dysregulation of the immune system in response to infection (often hyper-inflammatory, followed by anergy) and is associated with severe organ damage and failure with a rapid onset of mortality [57].

Early studies conducted by Carlson described a synergistic effect on mortality in a mouse model of *C. albicans*–*S. aureus* polymicrobial IAI [58]. The LD_50_ of the *C. albicans* strain was 2.9 × 10^8^ CFU (colony-forming unit); the LD_50_ of the *S. aureus* strain (2460, isolated from a patient with toxic shock syndrome) was determined to be 8 × 10^8^ CFU. Outbred CD-1 mice were infected intraperitoneally (i.p.) with *C. albicans* (7 × 10^6^ CFU), *S. aureus* (8 × 10^7^ CFU), or *C. albicans + S. aureus* at these same doses in saline. Results indicated that monomicrobial infection of either *C. albicans* or *S. aureus* was nonlethal, whereas dual infection with these sub-lethal doses of each organism caused nearly 100% mortality within three days post infection (d.p.i.). Further experiments indicated that the ideal ratio of *S. aureus*:*C. albicans* to display synergistic lethality was approximately 10:1. Additionally, heat-inactivation of either organism eliminated this apparent synergism [58]. These experiments helped develop initial concepts of synergistic lethality, whereby two microbes interact in a way that augments the virulence of one or both organisms, leading to enhanced morbidity and/or mortality.

Subsequent studies by Carlson evaluated the interaction between *C. albicans* and additional pathogenic bacteria, including *Serratia marcesans* and *Enterococcus faecalis* [59]. Interestingly, *C. albicans* was able to enhance virulence with all bacterial species tested, again using sub-lethal doses of both fungi and bacteria. Analysis of the blood, the pancreas, the kidney, and the spleen demonstrated that *E. faecalis* or *S. marcesans* burdens were nearly identical in the respective tissues, regardless of the initial dose when given along with a standard dose of *C. albicans*. It was also noted that the bacterial burden in mice infected with bacteria alone was undetectable, whereas *C. albicans* colonization was unaltered by the presence or the absence of bacteria. Thus, a major conclusion from this work was that, although *C. albicans* amplifies the virulence of other microbes, this synergism does not seem to be mutual, as the bacteria tested did not appear to affect the colonization or the virulence of *C. albicans* during polymicrobial IAI [59].

Carlson further defined the infectious relationship between *C. albicans* and *S. aureus* in murine polymicrobial IAI. Robust synergistic lethality was only observed when both pathogens were given i.p. [60]. Interestingly, when *C. albicans* was given i.p. and *S. aureus* was inoculated subcutaneously (s.c.), lethality was observed in 30% of animals, and mixed-infection was established only at the site of fungal inoculation. This suggests that *S. aureus* is migratory during infection and that *C. albicans* is needed for staphylococcal colonization to persist, perhaps due to some yet identified protective effect provided by the fungi [60]. Despite these important fundamental findings, the mechanism of synergistic lethality between *C. albicans* and *S. aureus* in polymicrobial intra-abdominal infection was left largely undefined until recently.

### 4.4. Mechanisms of Synergistic Lethality: a role for staphylococcal toxins

Decades following the groundwork laid by Carlson, a new study emerged providing insight into the pathogenesis of *C. albicans*–*S. aureus* IAI [61]. Similar to previous findings, intra-abdominal co-infection with *C. albicans* and *S. aureus* led to synergistic lethality with mice succumbing to infection by approximately 48 h p.i. Attention to the host response revealed significantly higher levels of neutrophils recruited to the peritoneal cavity during co-infection along with synergistic increases in cytokines Interleukin-6 (IL-6), Granulocyte-Colony Stimulating Factor (G-CSF), Keratinocyte Chemoattractant (KC), Monocyte Chemoattractant Protein-1 (MCP-1), and Macrophage Inflammatory Protein 1-α (MIP-1α) in the spleen and the kidneys, indicating robust inflammation. Interestingly, this study also revealed synergistic increases of the eicosanoid Prostaglandin E_2_ (PGE_2_) in the peritoneal lavage fluid. Prophylactic reduction of PGE_2_ using the cyclooxygenase inhibitor indomethacin significantly protected mice from co-infection and lowered inflammatory markers. Importantly, staphylococcal toxins, including ⍺- and δ-toxin, have been shown to activate phospholipase A2 signaling, leading to increased generation of prostaglandins [62,63].

In order to unravel the mechanism of interaction between these two important pathogens, one must consider the virulence determinants of both organisms as well as the host response in driving the synergistic lethality. For *C. albicans*, this includes the morphological switch from yeast to hyphae and adhesive factors. Interestingly, in contrast to the oral co-infection model, Als3p binding was found to have no influence on synergistic mortality during *C. albicans*–*S. aureus* polymicrobial intra-abdominal infection [64]. This finding was indirectly supported by Nash et al. in their investigation into the role of morphogenesis during *C. albicans*–*S. aureus* polymicrobial infection. This study showed that a yeast-locked *C. albicans* strain had no defect in promoting lethal synergism with *S. aureus* during polymicrobial IAI as compared to co-infection with a wild-type strain [65]. Additionally, a hypha-“locked” *C. albicans* strain did not show enhanced mortality as compared to wild-type infection [65]. Moreover, co-infection with a variety of non-*albicans Candida* species resulted in disparate infectious outcomes. Co-infection of *S. aureus* with *Candida krusei* (does not form hyphae) led to synergistic mortality similar to that of co-infection with *C. albicans*, while co-infection with *Candida dubliniensis* (close filamentous phylogenetic relative of *C. albicans*) was non-lethal [66]. These data indicate that the morphology of *C. albicans* is not required for its ability to enhance *S. aureus* virulence during IAI. Interestingly, co-infection with the hypha-defective *efg1*Δ/Δ/*cph1*Δ/Δ mutant failed to drive lethal synergism, indicating that expression of downstream target genes and not hyphal growth per se are important factors promoting infectious synergism [67].

As mentioned prior, *S. aureus* virulence is mainly due to toxin production and is intimately tied to QS. However, not all *S. aureus* strains have the same toxin profile, as major inter-strain heterogeneity with respect to genetic presence/absence and relative expression of toxin-producing genes is apparent [68,69]. Carlson used various *S. aureus* strains with different toxin profiles in her initial studies of *C. albicans*–*S. aureus* synergism, focusing on toxic-shock syndrome (TSS)-associated isolates and non-TSS disease-associated isolates [70]. Unsurprisingly, the majority of TSS-associated isolates were positive for toxic shock syndrome toxin (TSST), while the non-TSS isolates were negative. She found that polymicrobial infection in mice with *C. albicans* and TSS-associated *S. aureus* strains led to 100% mortality within 2 d.p.i., whereas non-TSS-associated strains caused 100% mortality much sooner, typically within 15 h.p.i. Due to the lack of genetic tools and isogenic strains at the time, further characterization of specific toxins was not possible, thus it is unknown which other toxin(s) played a role in these results [70]. Despite this, Carlson’s work using spent culture supernatants from *S. aureus* during co-infection clearly hinted that staphylococcal exotoxins contribute to synergistic lethality [71].

A recent study by Todd et al. determined that α-toxin is a major driver of lethality in *C. albicans*–*S. aureus* polymicrobial infection [64]. Transcriptional responses of several major *agr*-dependent genes as well as related protein expression levels were assessed during monomicrobial and polymicrobial growth with *C. albicans*. The data indicate that α-toxin (encoded by the *hla* gene) was highly upregulated in an *agr*-dependent fashion during co-culture. As α-toxin exhibited hemolytic activity, it produced zones of hemolysis on blood agar, which were used as readouts for α-toxin production. Hemolysis was enhanced when *S. aureus* was grown with *C. albicans*, and this was also quantified using an α-toxin specific ELISA. The strongest evidence for the crucial role of α-toxin in this specific interaction was the noted avirulence of an α-toxin null mutant (Δ*hla*) during co-infection with *C. albicans* in the mouse model of IAI. Complementation of *hla* restored the synergistic lethal phenotype. Strikingly, independent of bacterial burden, α-toxin was found to be elevated in the peritoneal cavity during co-infection as compared to monomicrobial infection; this was also correlated with synergistic elevation of PGE_2_ in the peritoneal cavity. Additional experimentation demonstrated that passive immunization of mice with the anti-α-toxin antibody MEDI4893* led to significant protection (>70% survival) during polymicrobial challenge [64]. Collectively, these data support the conclusion that α-toxin is the main staphylococcal effector driving lethality during *C. albicans*–*S. aureus* polymicrobial IAI and that *C. albicans* enhances *agr* signaling (Figure 3). Whether other *agr*-regulated staphylococcal toxins beside α-toxin play a role in governing infectious synergism remains to be determined.

## 5. Lessons Learned, yet Questions Remain

While significant progress regarding host signaling events (e.g., prostaglandins and elevated inflammation) and microbial interactions (e.g., augmentation of α-toxin) has been made to better delineate why *C. albicans* and *S. aureus* display infectious synergism during polymicrobial IAI, some important questions desperately require answers, such as: which candidal factor(s) drive increased *agr* signaling, why is this pathway up-regulated, and how exactly is the host succumbing to infection following co-infection?

While it is evident that α-toxin is augmented both in vitro and in vivo during growth with *C. albicans*, the mechanisms by which the toxin becomes up-regulated are still undefined. It is possible that *C. albicans* secretes a small peptide, whether QS-related or not, that engages the AgrC receptor on the surface of *S. aureus* similar to AIP2 to stimulate staphylococcal *agr* gene regulation. Experiments using a Δ*agrB* mutant that is unable to process AgrD into mature AIP2 but retains AgrC/AgrA expression and an otherwise intact *agr* circuit may be useful in teasing out this possibility. Another possibility is that fungal-driven altered environmental conditions could impact *agr* signaling. Expression of *agr*-regulated genes is surprisingly sensitive to pH; conditions that are too acidic or alkaline terminate *agr* signaling [72,73]. It has been recently shown that *C. albicans* can alkalinize the external environment through amino acid catabolism via secretion of ammonia into the extracellular space as waste [74,75]. As *S. aureus* tends to lower the pH via metabolism of glucose—creating acidic end products—it is possible that *C. albicans* could buffer the pH at a level maximal for *agr* activation. While this could potentially explain elevated levels of α-toxin in vitro, it would be difficult to reconcile this model with the tightly controlled pH of the peritoneal cavity. That said, *C. albicans* infections of the murine peritoneum tend to result in a slightly more alkaline global external environment [76]. Perhaps modulation of pH in microenvironmental niches could contribute to enhanced toxin activation.

As organisms that both largely asymptomatically colonize the human host, it would not be particularly advantageous to elicit such robust virulence and subsequent lethality. Thus, it is somewhat perplexing as to why *C. albicans* seemingly augments *S. aureus agr* signaling. It is conceivable that α-toxin could have an antagonistic effect on *C. albicans*, thus allowing for a competitive advantage during co-culture. Preliminary studies to address this in our laboratory using purified α-toxin neither inhibited *C. albicans* growth nor led to the uptake of propidium iodide to indicate fungal damage (unpublished data). However, α-toxin is not the only toxin up-regulated by the *agr* system; others, including δ-toxin and the PSMs, are also *agr*-regulated. Interestingly, *S. epidermidis* (a close relative of *S. aureus*) also utilizes a similar *agr* system and its δ-toxin demonstrates bacteriostatic effects on Group A streptococci [77], presumably conferring a selective advantage over other endogenous microbiota during skin colonization. Thus, it is possible that additional toxins have growth inhibitory effects on *C. albicans*. However, co-culture biofilm experiments using the Live/Dead staining system have revealed no apparent damage to hyphal cells [46]. However, *agr* activity was not specifically addressed in these experiments, necessitating further interrogation of this hypothesis.

Lastly, we still do not have a firm understanding of what is happening within the host that contributes to such striking synergistic lethality in such a small temporal window (as few as 16 h p.i. in some cases). While it is clear that inflammation and PGE_2_ signaling partially drive lethal outcome, α-toxin has other effector functions beside membrane damage, lysis, and eicosanoid stimulation. It can activate signaling through its high affinity host receptor a disintegrin and metalloprotease 10 (ADAM10), which ultimately leads to disruption of tight junctions and tissue desquamation on endothelial and epithelial cells [78]. α-toxin was also recently shown to activate platelet aggregation and dysregulate the hemostatic system, resulting in excessive clotting and liver injury due to exacerbated thrombosis [79]. Interestingly, the MEDI4893* antibody that was protective against lethal challenge during polymicrobial IAI also prevents such thrombotic events in the liver. Thus, it is conceivable that dysregulated hemostasis, mediated by elevated levels of α-toxin followed by subsequent organ failure, contributes to the synergistic mortality observed during polymicrobial IAI with *C. albicans* and *S. aureus*. Therefore, modulation of the clotting cascade may confer some level of protection during co-infection. While recent studies have largely focused on specific mechanisms (e.g., toxin, PGE_2_) driving lethal synergism, the use of unbiased approaches such as host–pathogen transcriptomic sequencing, metabolomics, and proteomics may yield novel insights into both microbial and host factors governing disease pathogenesis moving forward.

## 6. Conclusions

While we have gained significant insight into the complex relationship existing between *C. albicans* and *S. aureus*, there is still much to learn regarding this fascinating microbial pair. As sequencing technologies become less expensive and more sensitive, it is imperative to understand how microbiome, metagenome, and immune system shape these interactions within the human host, so that optimal and targeted therapies can be devised. Along with others, these case studies regarding *C. albicans*–*S. aureus* interactions have allowed the microbiology field to peer beyond the monomicrobial paradigm that Koch proposed a century before and have helped usher in a new frontier. If this much information was gleaned from a single fungal–bacterial pairing, exciting discoveries on the horizon regarding additional microbial interactions are virtually endless.
